# Stapled Hemorrhoidopexy: “Mucosectomy or Not Only Mucosectomy, This Is the Problem”

**DOI:** 10.3389/fsurg.2021.655257

**Published:** 2021-03-12

**Authors:** Chiara Eberspacher, Fabio M. Magliocca, Stefano Pontone, Pietro Mascagni, Lisa Fralleone, Gaetano Gallo, Domenico Mascagni

**Affiliations:** ^1^Department of Surgical Sciences, University of Rome ‘Sapienza’, Rome, Italy; ^2^Department of Radiological Sciences, Oncology, and Pathological Anatomy, University of Rome ‘Sapienza’, Rome, Italy; ^3^IHU-Strasbourg, Institute of Image-Guided Surgery, Strasbourg, France; ^4^Fondazione Policlinico Universitario A. Gemelli IRCCS, Rome, Italy; ^5^Department of Medical and Surgical Sciences, University of Catanzaro, Catanzaro, Italy

**Keywords:** stapled hemorrhoidopexy, rectal mucosectomy, histopathology, complications, full thickness

## Abstract

**Introduction:** Stapled hemorrhoidopexy was originally defined as a rectal mucosectomy. The aims of our retrospective, single-center study were to demonstrate if the excised specimen comprises only the mucosa or more wall rectal layers and if the latter excision should be considered a technical mistake with an increase in complications.

**Materials and Methods:** We histopathologically analyzed surgical samples from patients who underwent stapled hemorrhoidopexy performed between 2014 and 2019. Patients were divided into three groups, according to the stapler used: Group A (single PPH®), Group B (double PPH®), and Group C (CPH34 HV™). We evaluated the actual wall layers included in the stapled rectal ring. For every specimen, we reconstructed the history of the corresponding patient and the incidence of complications.

**Results:** Of the 137 histological slides available, 13 were only mucosectomies (9.5%), and 124 presented also the submucosa and muscularis propria (90.5%)−50/58 patients in Group A, 28/28 in Group B, and 46/51 in Group C. No statistically significant difference in the rate of complications was found when stratifying patients according to the thickness of the resection [mucosectomy (M) or “full thickness” (FT)].

**Discussion:** Stapled hemorrhoidopexy is not a simple mucosectomy but a resection of the rectal wall with almost all its layers. This concept defines the entity of the surgical procedure and excludes a direct correlation with an increased rate of complications.

## Introduction

Hemorrhoidal disease (HD) is a common proctologic disease, characterized by enlarged, inflamed, thrombosed, or prolapsed hemorrhoids, with symptoms like pain and rectal bleeding. Prevalence of HD changes according to studies and criteria of definition, from 25 to 39% in adult population (1).

The procedure for prolapse and hemorrhoids (PPH) was introduced in 1993 as novel treatment for HD and was originally described as a rectal mucosectomy (2). The procedure gained in popularity thanks to its creator Antonio Longo, who, in his report on this stapled hemorrhoidopexy (SH) (3), described the object of the excision as a rectal internal mucosal prolapse. The first stapler used as a dedicated device for this procedure was PPH; to remove more tissue, in an attempt to reduce recurrences, two PPH or high volume instruments—CPH34 HV—with a bigger case were used. Today, many articles talk about “mucosectomy” when referring to SH (4). At the same time, many life-threatening complications, such as perforations, vascular lesions, or hematomas, that are connected with these procedures are often ascribed to a presumed uncorrected “more than mucosa” resection (5). The aim of the present study is to evaluate, during SH, despite the different techniques and tips or tricks of the surgeons, if the stapled ring include only the mucosa or it is a “full-thickness” excision and if the latter feature should be considered a technical mistake, increasing the complication rate.

## Methods

This retrospective, observational, single-center study is reported according to the Strengthening the Reporting of Observational Studies in Epidemiology (STROBE) statement for cohort studies (6). It included samples derived from SH, performed in the Department of Surgical Sciences of our hospital from 2014 to 2019. We enrolled patients, more than 18 years old, who had undergone surgery for hemorrhoidal disease (grade III and IV hemorrhoids or grade II when symptomatic) (7, 8). Exclusion criteria were an association with other anorectal disease (anal fissure, fistula, and perianal diseases), inflammatory bowel disease, chronic therapy with anti-inflammatory drugs, the presence of incontinence, and previous operations for hemorrhoids or prolapse. We also excluded patients who had undergone operations performed by residents to minimize bias due to the lack of experience in the procedure; all surgeons had an experience of at least 50 SH before the lapse of time considered for the study. All patients gave informed consent for surgery and histological examination of the specimen.

Patients were divided into three groups, according to the different stapler devices used, to determine whether a different stapler could change the entity of the resection: Group A underwent operations performed with a single PPH® stapler, Group B with a double PPH®, and Group C with a CPH34 HV™. All operations were performed in lithotomic position under spinal or general anesthesia, and the average hospital stay was 2 days. The rectal ring was obtained, realizing a purse-string suture in all cases. We excluded all the patients treated by SH when we used a “parachute technique,” in order to remove more tissue. All stapler rings were fixed in formalin, with no orientation, and arranged on histological slides. The preserved histological slides were re-analyzed by a pathologist (FM) to evaluate the different layers—the presence of only the mucosa (M) or even of the submucosa and muscularis propria (FT). The expert was blinded to the previous description of the specimen.

We traced the patient and their clinical history and any minor complications, including pain evaluated with a Visual Analog Scale (VAS; ranging from 0 to 10, with 10 being full pain and 0 no pain), incontinence evaluated with the Wexner score (WS), time to return to work, and persistent urgency. Data were collected in our prospective PC database. We also evaluated any major complications, including sepsis, bleeding requiring new hospitalization, hematoma, and recurrence in the first year of follow-up. Follow-up consisted of outpatient visits with digital rectal exploration (after 1 week, 1 month, 3 months, and 6 months), with a clinical control consisting of a rectal digital examination and a questionnaire about symptoms. This was followed by a phone call after 1 year, with a further outpatient visit if necessary. The presence of recurrences was also determined by clinical examination.

The definition of the true wall layers of the rectal rings excised during these operations, despite the common use of the term “mucosectomy” in the literature (7), was the main aim of our study. A secondary endpoint was to evaluate if the thickness of the resected specimen was correlated with the incidence of complications and was tested by dividing all the patients into two groups: the M Group (mucosectomy) and the FT Group (“full thickness”).

Data were analyzed using SPSS for Windows, version 21 (SPSS Inc., Chicago, IL). Means and standard deviations were used to report continuous data, while numbers and percentages were calculated for all categorical data. Univariate analysis was performed with Student's *t*-test. *p* ≤ 0.05 was considered statistically significant for all analyses.

## Results

We identified a total of 304 SH procedures performed in our Department of Surgical Sciences of “Sapienza” University of Rome that met our inclusion criteria. Only 137 had histological slides available for re-examination. Of those, 124 belonged to female patients (90.5%) and 13 to male patients. The mean patient age was 52 years (range: 21–83 years).

**Figure 1:**
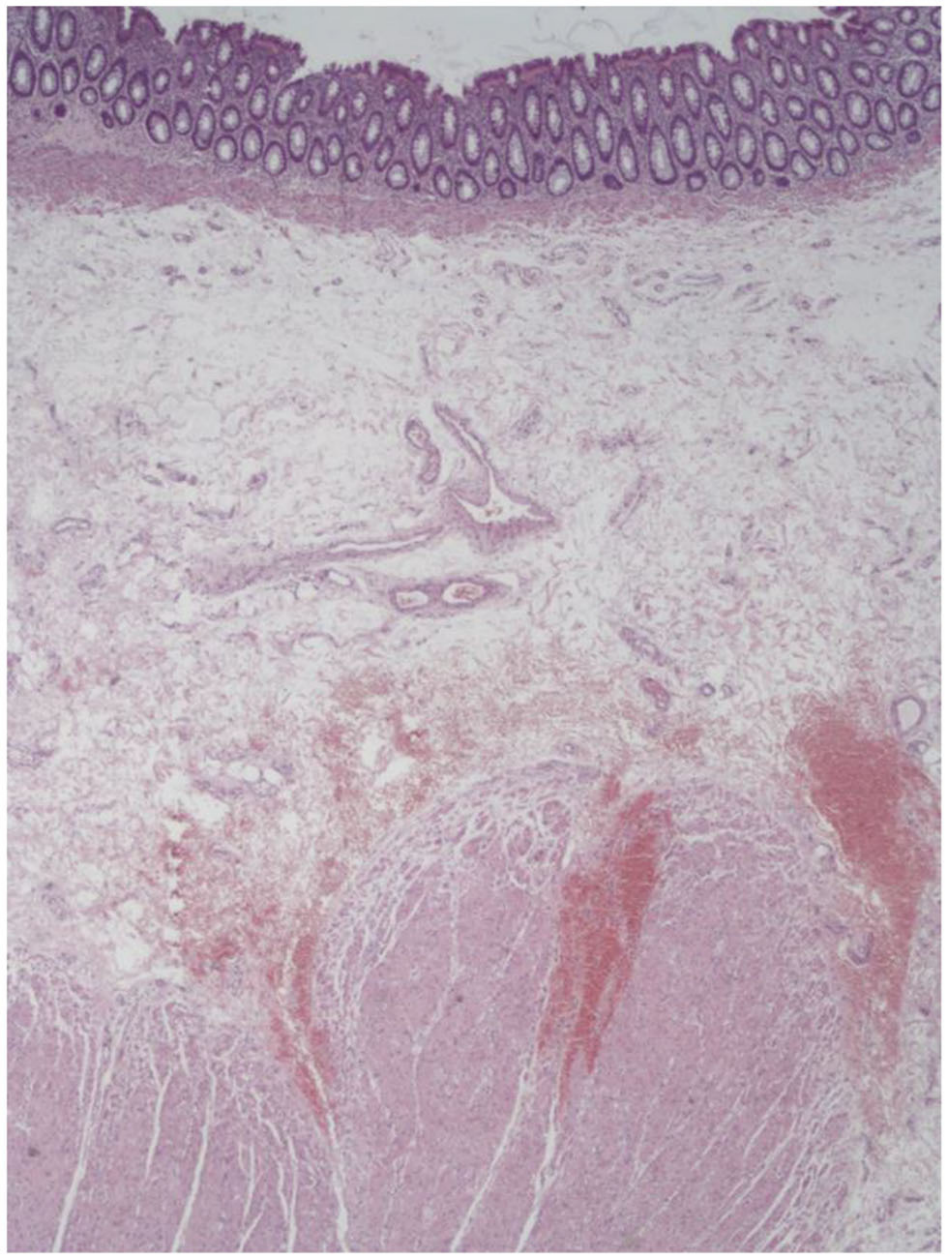
An example of stapled hemorrhoidopexy (SH) specimen: well visible are the mucosa, submucosa, and muscularis propria.

**Figure 2:**
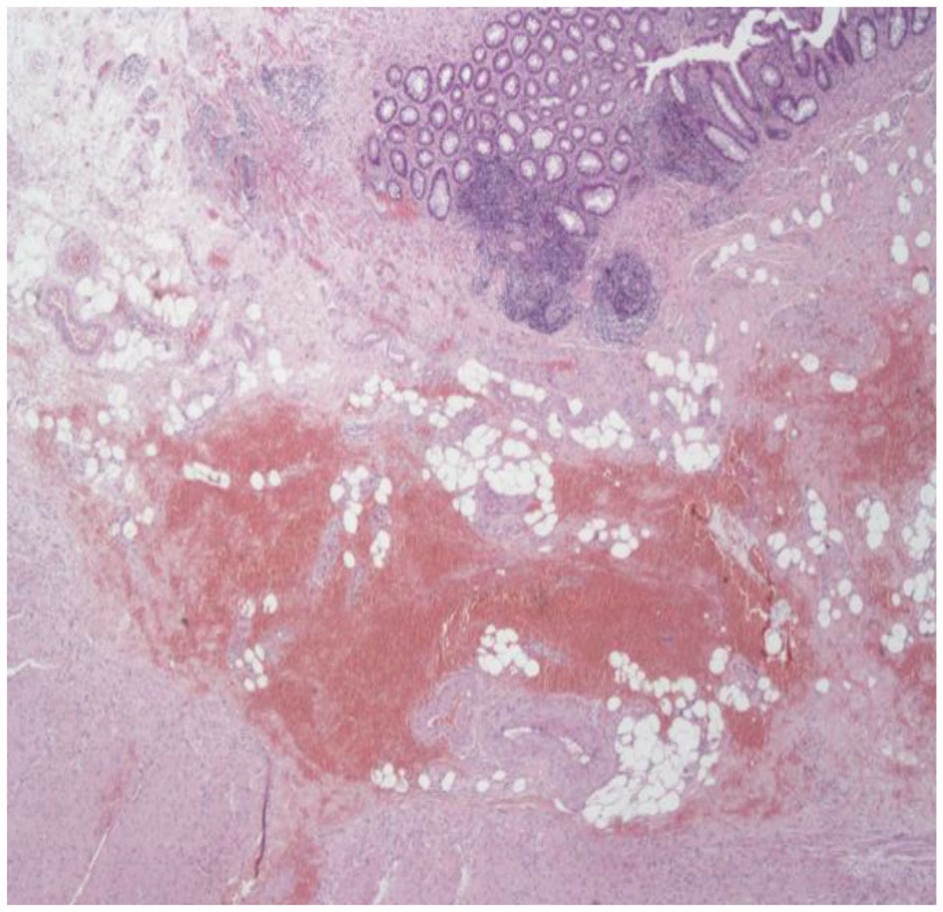
Intramural bleeding in the submucosa of a specimen.

Group A (single PPH®) consisted of 58 patients, Group B (double PPH®) 28 patients, and Group C (CPH34 HV™) 51 patients. Of those, we identified a true mucosectomy in eight patients (13.7%) in Group A, none in Group B (0%), and five (9.8%) in Group C (Table 1). All the patients in most of the cases (124/137: 90.5%) had a histological slide that included the mucosa, submucosa, and tunica muscularis (Figure 1). The anal ring thickness was about 8–10 mm: in 11 cases, a submucosa thicker for an intramural bleeding does not permit to include in the stapler case all the muscularis propria, and only some muscular fibers were found (partial muscularis propria resection) (Figure 2).

**Table 1:** Histopathological study of the rectal ring, after SH (137 patients).

	**Group A (single PPH)**	**Group B (double PPH)**	**Group C (CPH34/CPH36)**
Mucosectomy only	8	0	5
Full thickness, with muscularis propria	50	28	46
Total of patients	58	28	51

*SH, stapled hemorrhoidopexy; PPH, procedure for prolapse and hemorrhoids*.

The patients with pure mucosectomy were almost all male with a rate of 53.8% (7/13). Only 4.8% (6/124) of the female patients had no submucosa and muscular fibers in the specimen.

Stratification of the patients according to the thickness of the resection (the M Group vs. group FT) and considering minor complications revealed no statistically significant differences. We observed 13 patients in Group M and 124 in Group FT: mean VAS after first week was 3.6 (SD 1.68) in Group M vs. 3.7 (SD 1.69) in Group FT (*p* = 0.33), mean WS after 1 month 1.8 (SD 1.8) in Group M vs. 2.3 (SD 2) in Group FT (*p* = 0.22); the date of return to work was similar [post-operative (PO) day 9 SD 2.6 in Group M vs. 8.6 SD 2.9 in Group FT; *p* = 0.26]. Two patients in the FT Group (one in Group B and one in Group C) reported urgency and a persistent increase in stool frequency, but these complications resolved after 4 and 6 months.

Reports of major complications were very rare: one case in the FT Group (Group A; single PPH) experienced an episode of post-operative bleeding on the fourth PO day and required a new hospitalization with an evaluation under anesthesia and surgical hemostasis. No cases of perirectal hematoma or sepsis occurred. Only in one case did we observe an early recurrence of the hemorrhoidal disease; this was in a male patient in the M Group (Group A) at 8 months (Table 2).

**Table 2:** Minor and major complications after SH—mucosectomy vs. full thickness.

**Complications**	**M Group (mucosectomy) 13 pts**	**FT Group (full thickness)124 pts**	***p***
Pain (VAS)Mean (SD)	3.6 (1.68)	3.7 (1.69)	0.33
Incontinence (WS)Mean (SD)	1.8 (1.8)	2.3 (2)	0.22
Return to work (PO days)Mean (SD)	9 (2.6)	9.5 (2.9)	0.26
Major bleeding (pts)	0	1	0.38
Urgency (pts)	0	2	0.32
Recurrences (pts)	1	0	-

*SH, stapled hemorrhoidopexy; VAS, Visual Analog Scale; WS, Wexner score*.

## Discussion

Treatment of HD by SH is a widespread technique, but since its initial use, it has often been described as a stapled mucosectomy. We conducted a thorough review of the literature using the keywords “mucosectomy” and “hemorrhoids” on PubMed in December 2020 and found six papers among the most recent 40 studies (5, 9–13) that used the term mucosectomy to describe the type of surgery adopted to treat hemorrhoids. We found no prevalence for any particular country or journal: from China to Italy, when a stapler is used to reduce a hemorrhoidal prolapse, the common term used is mucosectomy.

Unfortunately, despite the large number of studies dedicated to the evaluation of follow-up, complications, and costs, there are still no studies that focus on the characteristics of the tissue removed during surgery. Pathology examinations of hemorrhoids and prolapse specimen can, in some cases, reveal incidental findings (14), but the majority of the reports describe the anal ring generically, as a portion of rectal wall, with no further specification. We decided to do a new examination with the aim of providing a more accurate description of the different rectal wall layers by characterizing actual tissue excised during surgery. In most cases in which the excised tissue allowed a histological re-evaluation, we were faced with a “full-thickness” resection that included, in addition to the mucosa, also the submucosa and the muscularis propria. Only 13/137 specimen (9.5%) were true mucosectomies.

In the majority of cases, an actual mucosectomy was performed only in male patients. This is not surprising considering the characteristics of the mucosal prolapse associated with hemorrhoids, as this prolapse occurs more frequently in women (15) and, in these cases, appear more mobile than in male patients. It is common, in female patients, to include a greater amount of tissue in the stapler case.

If the first endpoint of the study is fundamentally anatomical and to define the entity of the excision, the second endpoint is clinically even more important: it is to evaluate if this type of resection (FT) increases complications. In the past, some studies have reported, for example, an increase in post-operative pain and fecal urgency after stapled hemorrhoidectomy (16), and this has been interpreted as a consequence of incorporation of muscle fibers in the doughnut specimen. Hidalgo et al. (17) reported a perirectal hematoma after a resection that included not only the mucosa but also the submucosa, muscular layer, and perirectal fat tissue. Our observations of common specimens obtained after SH indicate that to remove more than mucosa seems to be the rule (Figure 1).

A full thickness resection of a prolapse did not increase complications. Even though dividing the patients into two groups according to the type of resection (the M Group and the FT Group) did not allow a valid statistical comparison because of the different numbers of patients (13 vs. 124), we can still make some suppositions. In the FT Group, the number of complications was not significantly higher than in the M Group, but it was also not higher than in the general reports about complications after SH. Relevant bleeding occurred in only 1/124 patients (0.8%), which was less than that reported in other studies (18). Persistent urgency, without any serious episodes of incontinence, was present in two cases, both in the FT Group (1.6%). The only early recurrence (before 1 year of follow-up) was found in one patient, in the M Group (7.7%).

The small sample size, however, limited the statistical power of the analysis carried out to evaluate the complication rate in relation to the thickness of the removed rings.

In the present study, we histopathologically evaluate the actual wall layers excised during SH, in order to define if the use of the term “mucosectomy” is appropriate. From the beginning of the technique until the present, this has been a “popular” definition. Some surgeons assumed that SH was not only a mucosectomy; others declared that a full thickness excision should be considered a technical mistake, related to post-operative complications (19).

This study demonstrated that this is not only a mucosectomy, but it is actually a true resection of the rectal wall—analysis of the specimens confirmed the presence of muscular fibers in 90% of the cases. Further, this study excluded a correlation between the rate of post-operative complications, mild and severe and a “full thickness” resection. This is almost the rule in SH and not a surgical accident.

## Data Availability Statement

The raw data supporting the conclusions of this article will be made available by the authors, without undue reservation.

## Ethics Statement

Ethical review and approval was not required for the study on human participants in accordance with the local legislation and institutional requirements. The patients/participants provided their written informed consent to participate in this study.

## Author Contributions

CE and DM designed the study, analyzed the data, wrote the manuscript, designed tables, and gave final approvation. FM analyzed the data, realized the pictures, and gave final approvation. SP and GG analyzed the data, corrected the final draft, gave an important contribue to the final draft, revised bibliography, and final approvation. PM and LF collected the data, improved quality of pictures, and gave final approvation. All authors contributed to the article and approved the submitted version.

## Conflict of Interest

The authors declare that the research was conducted in the absence of any commercial or financial relationships that could be construed as a potential conflict of interest.

## Abbreviations

HD: hemorrhoidal disease
PPH: procedure for prolapse and hemorrhoids
CPH: circular stapler for rectal prolapse and hemorrhoids
SH: stapled hemorrhoidopexy
VAS: Visual Analog Scale
WS: Wexner score
PO: post-operative.
